# Impact of previous pregnancy and BMI on cellular and serum immune activity from early to late pregnancy

**DOI:** 10.1038/s41598-024-66651-4

**Published:** 2024-07-11

**Authors:** Grace Mealy, Kiva Brennan, Sarah Louise Killeen, Mark Kilbane, Cara Yelverton, Radka Saldova, David Groeger, Douwe VanSinderen, Paul D. Cotter, Sarah L. Doyle, Fionnuala M. McAuliffe

**Affiliations:** 1 UCD Perinatal Research Centre, School of Medicine, University College Dublin, National Maternity Hospital, Dublin 2, Ireland; 2 Department of Clinical Medicine, Trinity College Institute of Neuroscience, School of Medicine, Trinity College Dublin, Dublin 2, Ireland; 3 Department of Clinical Chemistry, St Vincent’s University Hospital, Dublin, Ireland; 4 The National Institute for Bioprocessing, Research, and Training (NIBRT), Dublin, Ireland; 5 UCD School of Medicine, College of Health and Agricultural Science (CHAS), University College Dublin (UCD), Dublin, Ireland; 6 PrecisionBiotics Group Ltd (Novozymes), Cork Airport Business Park, Kinsale Road, Cork, Ireland; 7 School of Microbiology, University College Cork, Cork, Ireland; 8 APC Microbiome Ireland, University College Cork, Cork, Ireland; 9 Moorepark, Teagasc Food Research Centre, Fermoy, Cork, Ireland

**Keywords:** Adaptive immunity, Antimicrobial responses, Cytokines, Inflammation, Innate immunity, Lymphocytes, Immunology, Biomarkers

## Abstract

Immunological adaptions during pregnancy play a crucial role in healthy fetal development. Aberrant immune modifications however contribute to adverse pregnancy outcomes, which may be driven by maternal factors such as previous pregnancies and BMI. This secondary analysis of the MicrobeMom2 RCT investigates the changes to maternal inflammatory biomarkers derived from serum and stimulated peripheral blood mononuclear cells (PBMCs) during pregnancy, and the effects of previous pregnancies (parity) and BMI on maternal immune responses. Changes in immune and metabolic biomarkers from early (11–15 weeks’ gestation) to late (28–32 weeks’ gestation) pregnancy were compared using paired t-tests. Participants were then split by parity (nulliparous, parous) and BMI (BMI < 25, BMI > = 25), and the relationship between parity and BMI with immune biomarker levels was examined using independent t-tests, paired t-tests, ANCOVA, and linear regression. Equivalent non-parametric tests were used for skewed data. Recruited women (n = 72) were on average 31.17 (SD ± 4.53) years of age and 25.11 (SD ± 3.82) BMI (kg/m^2^). Of these, 51 (70.8%) had a previous term pregnancy. Throughout gestation, PBMC cytokines displayed contrasting trends to serum, with a dampening of immune responses noted in PBMCs, and enhanced production of cytokines observed in the serum. Significant decreases in PBMC derived TNF-α, IL-10 and IFN-γ were seen from early to late pregnancy. Serum C3, IL-17A, IL-6, TNF-α, CD163, GDF-15 and leptin increased throughout gestation. First pregnancy was associated with higher levels of leptin in late pregnancy, while parous women showed significant decreases in PBMC derived TNF-α, IL10, and IFN-γ with gestation. Differences in levels of C3, IL-17A, TNF-α, GDF-15 and leptin were observed across BMI groups. Overall, serum-derived cytokines exhibit contrasting levels to those derived from stimulated PBMCs. Maternal immune responses undergo significant changes from early to late pregnancy, which are influenced by parity and BMI. These differences aid our understanding as to why first-time mothers are at greater risk of placental disease such as pre-eclampsia and fetal growth restriction.

## Introduction

The maternal immune system is faced with a unique complexity during pregnancy given that it must support the growth of a semi-allogenic fetus whilst also providing defence against infection^1^. This challenging task is facilitated by a series of essential immunological adaptations that occur from implantation to delivery^2^. Loss of immunological balance however can result in adverse pregnancy outcomes such as pre-eclampsia, pre-term birth and miscarriage^3^. Further study of both the normal maternal immune response and the factors which impact it is required to fully understand the driving factors of these immune imbalances.

Cytokine levels throughout pregnancy have been previously explored in the plasma and serum^4–9^. While many of these studies agree that pregnancy is a time of immunological change, there are contrasting reports of cytokine secretion patterns, likely due to differences in timing and methodology^3^. Cellular activity in the periphery has also been examined, which provides insight into the ability of cells to respond to infection^1,10–12^. However, patterns of cellular cytokine expression have also contrasted between studies, and few have reported immune component levels from both serum and peripheral blood mononuclear cells (PBMCs) in the same cohort^9^.

Discrepancies between studies may also be attributed to maternal factors. Some research has suggested that parity or body mass index (BMI) impacts immune activity. Pre-eclampsia, an inflammatory complication of pregnancy, has been shown to have increased prevalence in nulliparous women^13,14^. It is postulated that this is due to tolerability of the maternal immune system to paternal antigens in previous pregnancies^15^. Another risk factor for pre-eclampsia is high maternal BMI, which is believed to increase inflammation through the production of immune mediators in adipose tissues^16^. Elevated levels of circulating inflammatory proteins such as TNF-α, IL-6, and CRP have also been reported in mothers with overweight or obesity^17,18^.

To date, research on the impact of maternal parity and BMI on immune activity has been limited to the plasma or serum^4,5^. In addition to this, given the discrepancies that lie between studies of circulating and stimulated cytokines longitudinally in pregnancy, a comprehensive analysis of cytokines from both sources in one cohort is warranted. This may provide further understanding of normal pregnancy and disease.

In this secondary analysis of the MicrobeMom2 study (an RCT which included a probiotic treatment group and a control group), we examine the immunological changes that occur longitudinally from early (11–15 weeks’ gestation) to late (28–32 weeks’ gestation) pregnancy in both serum and PBMCs. An established panel of immune markers including PBMC derived IL-2, IL-6, IL-10, TNF-α and IFN-γ, and serum derived IL-17A, IL-6, TNF-α, and Leptin were analysed. In addition, a selection of more scarcely reported markers ICAM1, GDF-15 and CD163 were also investigated. This panel provides insight into inflammatory responses (IL-2, IL-17A, IL-6, TNF-α, IFN-γ, ICAM1), anti-inflammatory responses (IL-10, CD163, GDF-15), leukocyte adhesion (ICAM1) and metabolism (GDF-15, leptin). Immune differences were also compared between women with BMI < 25 and BMI > = 25, and nulliparous and parous women both longitudinally, and at early and late timepoints to further understand the impact of BMI and parity on maternal immune responses.

## Results

### The MicrobeMom2 RCT

The primary study, a randomised controlled trial of probiotic supplementation with *Bifidobacterium longum* 1714® versus placebo for a 12-week period during pregnancy, showed no difference in cytokine production between intervention and control groups. Further detail is available in the primary study publication^19^.

### Demographic and anthropometric data

All participants from the MicrobeMom2 primary study were included in this secondary analysis. Study participants (n = 72) were on average 31.17 (4.53) years of age. Sixty women (83.3%) were white Irish, and 51 (70.8%) were parous. No significant difference was observed between nulliparous and parous groups in terms of gestational age in days (279.91 (9.92) *vs.* 279.31 (9.20), p = 0.809). The average BMI of the study cohort in early pregnancy was 25.11 (3.82) kg/m^2^, with 38 women (52.8%) having a healthy BMI, and 34 (47.2%) having overweight or obesity. As defined by the World Health Organisation, a BMI of 18.5–24.9 is classed as healthy, BMI of ≥ 25 indicates overweight, while BMI of ≥ 30 indicates obesity^20^. 2 participants were diagnosed with gestational diabetes. No women experienced pre-eclampsia or pre-term birth. Of the 72 infants born, 39 (54.2%) were female, and 2 had macrosomia. Further results on the pregnancy and infant outcomes are available in the primary study publication^19^.

### Gestational changes in immune activity

**Figure 1:**
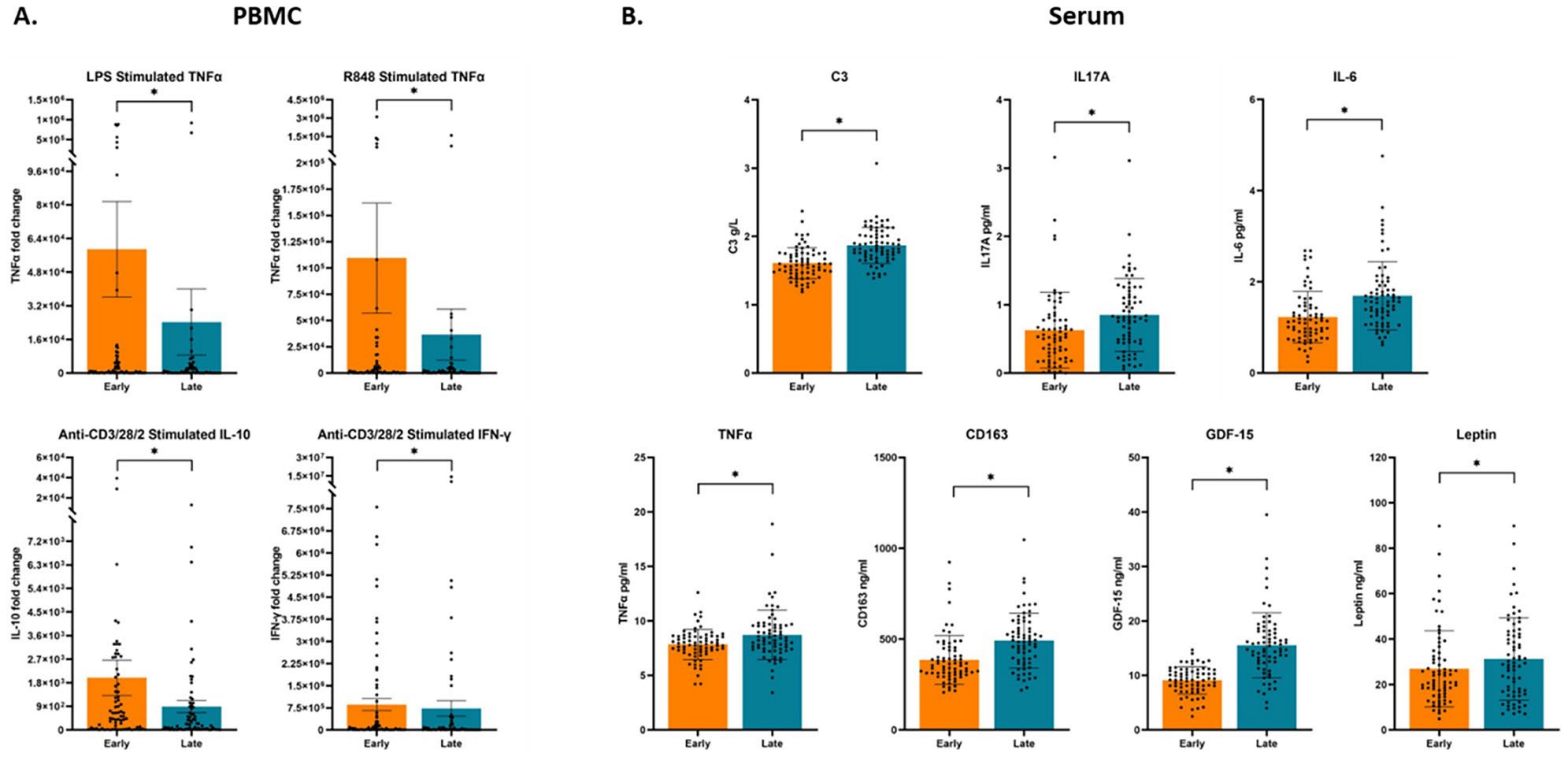
Serum and PBMC derived immune marker levels in early and late pregnancy. (**A**) Fold change levels of cytokines derived from PBMCs in early and late pregnancy. Significant findings from the paired t-tests conducted in Table 1 are shown, outlining significant differences in PBMC cytokines from early to late pregnancy. PBMCs were stimulated with either lipopolysaccharide (LPS), resiquimod (R848), or anti-CD3/28/2. (**B**) Biomarker concentrations of serum samples from early and late pregnancy. Significant findings from the paired t-tests conducted in Table 1 are shown, outlining significant differences in serum cytokines from early to late pregnancy. The data is presented as mean ± SEM, with each point corresponding to the individual biomarker concentration (serum) or fold change value (PBMC) for each mother. *Significant after Benjamini Hochberg adjustment for multiple comparisons, where the threshold for significance is defined as p < q.

When examining the changes in PBMC derived cytokines and serum markers from early (11–15 weeks’ gestation) to late (28–32 weeks’ gestation) pregnancy using paired t-tests, a significant decrease was observed in fold change levels of PBMC derived anti-CD3/28/2 stimulated IL-10 and IFN-γ, and LPS and R848 stimulated TNF-α, (Table 1,.Fig. 1A**).** No significant differences were observed in the fold change levels of IL-6 or IL-2 from early to late pregnancy after stimulation with LPS and Anti-CD3/28/2, respectively. In addition to fold change data, absolute values in pg/ml are provided in Supplementary Table S1. IFN-γ was the only unstimulated cytokine that did not change significantly from early to late pregnancy. Raw values of TNF-α, IL-10 and IL-2 decreased with gestation, while IL-6 increased. All raw stimulated cytokine concentrations decreased significantly with throughout pregnancy. In contrast to PBMC cytokines, an increase in immune activity was observed in serum (Table 1). From early to late pregnancy, concentrations of C3, IL-17A, IL-6, TNF-α, CD163, GDF-15 and leptin significantly increased, although by a marginal amount (Fig. 1B). No significant changes were identified in levels of CRP and ICAM1 between early and late pregnancy. Overall, cellular immune activity was suppressed, while immune components of the serum were enhanced from early to late pregnancy.

**Table 1:** PBMC and Serum Biomarkers from early to late pregnancy.

	Units	Stimulation *stimulant (incubation time)*	Early pregnancy *median (IQR)*	Late pregnancy *median (IQR)*	p-value
**PBMC**					
**IL-6**	Fold change	LPS (24h)	107.51 (8.91, 1133.23)	102.99 (9.66, 812.37)	0.508^a^
		R848 (24h)	310.36 (7.66, 1378.35)	159.42 (11.50, 616.88)	0.880^a^
**TNF-α**	Fold change	LPS (24h)	700.61 (38.47, 5310.09)	196.72 (8.59, 2427.23)	**0.016**^b^*
		R848 (24h)	1031.84 (80.61, 6481.81)	328.77 (20.80, 3092.72)	**0.017**^b^*
**IL-10**	Fold change	Anti-CD3/28/2 (48h)	519.89 (54.42, 1948.57)	183.06 (30.01, 887.03)	**0.014**^a^*
**IL-2**	Fold change	Anti-CD3/28/2 (48h)	13726.92 (3706.90, 32755.47)	6003.64 (1071.50, 43697.37)	0.946^a^
**IFN-y**	Fold change	Anti-CD3/28/2 (48h)	43531.70 (9374.35, 560538.75)	15281.12 (864.06, 183596.25)	** < 0.001**^a^*
**Serum**					
**C3**	g/l	*–*	1.59 (1.46, 1.75)	1.83 (1.71, 2.02)	** < 0.001**^a^*
**CRP**	mg/l	–	3.12 (1.90, 5.36)	3.41 (2.15, 5.31)	0.190^b^
**IL-17A**	pg/ml	–	0.53 (0.23, 0.80)	0.81 (0.46, 1.16)	** < 0.001**^a^*
**IL-6**	pg/ml	–	1.13 (0.82, 1.47)	1.56 (1.21, 1.92)	** < 0.001**^b^*
**TNF-α**	pg/ml	–	7.86 (7.23, 8.59)	8.41 (7.43, 9.60)	** < 0.001**^a^*
**CD163**	ng/ml	–	354.24 (307.64, 438.73)	486.60 (382.94, 589.26)	** < 0.001**^a^*
**ICAM1**	ng/ml	–	313.64 (281.25, 339.07)	299.28 (251.15, 352.26)	0.073^c^
**GDF-15**	ng/ml	–	9.17 (7.53, 10.86)	14.86 (12.38, 18.14)	** < 0.001**^a^*
**Leptin**	ng/ml	–	21.83 (15.52, 32.43)	29.14 (16.71, 42.13)	**0.005**^b^*

Paired t-test of fold change values and serum marker concentrations from early to late pregnancy. Fold change was calculated following stimulation of PBMCs with either lipopolysaccharide (*LPS*), Resiquimod (*R848*), or Anti-CD3/28/2 (*T-cell activator*) for 24 or 48 hours. n = 72.

^a^Wilcoxin Signed Rank Test on abnormally distributed data.

^b^Paired t-test on normally distributed log transformed data.

^c^Paired t-test on normally distributed data.

*Significant after Benjamini Hochberg adjustment for multiple comparisons.

### Impact of parity status on immune activity

**Figure 2:**
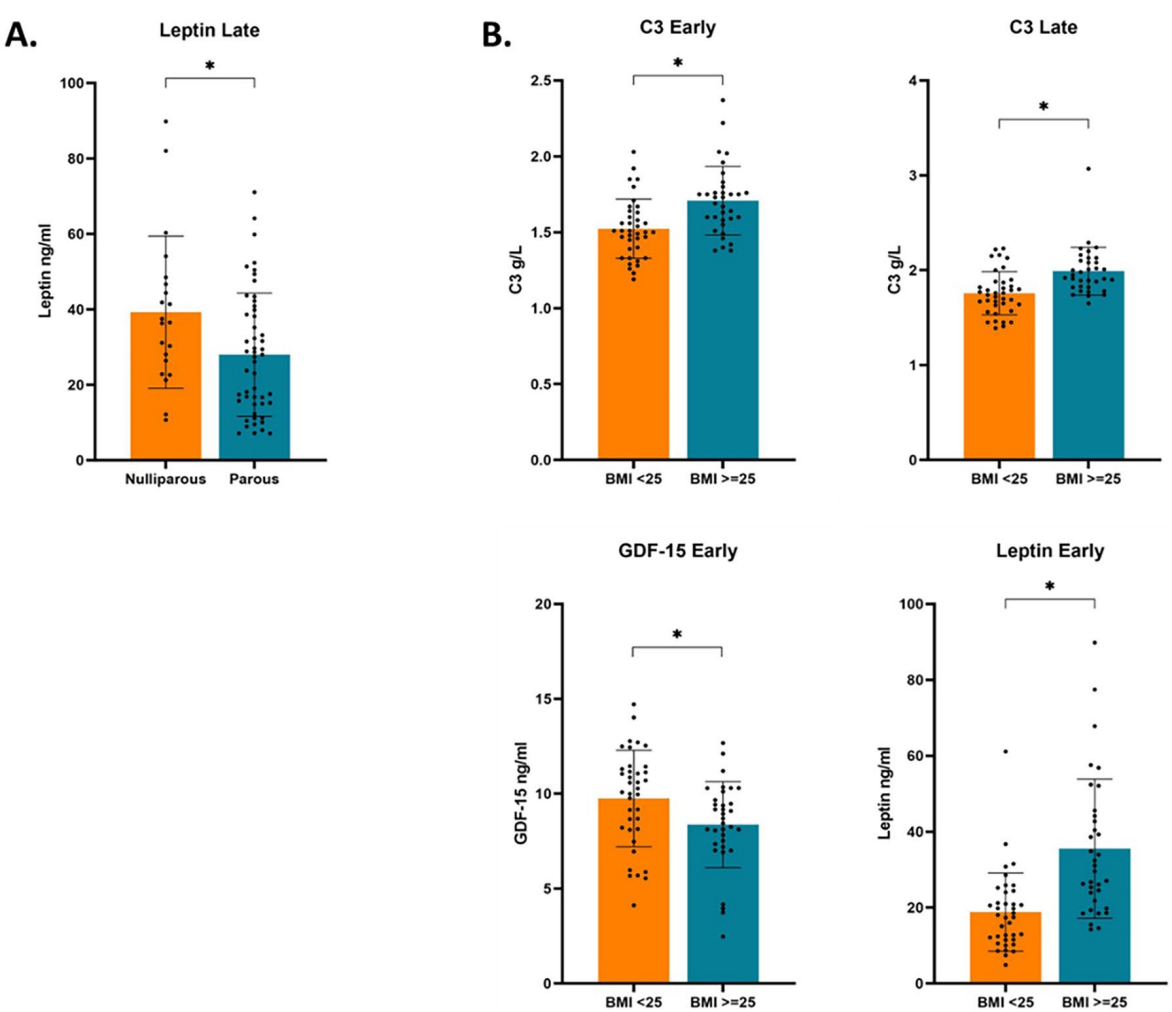
Differences in serum immune marker concentrations between parity groups and BMI groups. Significant differences in serum immune marker concentrations between parity groups and BMI groups. (**A**) Difference in late pregnancy serum leptin concentrations compared between nulliparous (n = 21) and parous (n = 51) mothers as determined by Analysis of covariance controlled for baseline (early) levels. Values are shown in Table 3. (**B**) Serum biomarker concentrations in either early or late pregnancy compared between mothers with a baseline BMI of less than 25 (n = 38), or greater than or equal to 25 (n = 34). Differences in early concentrations between groups were determined by independent t-test for normally distributed variables, or mann–whitney U test for non-parametric variables. Differences in late concentrations between groups were determined by Analysis of covariance controlled for baseline (early) levels. Values are shown in Table 5. Biomarkers with significant differences between BMI groups are shown. The data is presented as mean ± SEM, with each point representing the individual biomarker concentration for each mother. *Significant after Benjamini Hochberg adjustment for multiple comparisons, where the threshold for significance is defined as p < q.

Gestational changes explored through paired t-tests or Wilcoxon Rank sum tests yielded varying results between parity groups. In contrast to nulliparous women, from early to late pregnancy parous women showed a significant decrease in R848 stimulated TNF-α, anti-CD3/28/2 stimulated IL-10, and anti-CD3/28/2 stimulated IFN-γ from early to late pregnancy (Table 2). No significant differences were observed between late pregnancy values accounting for early values through ANCOVA after correction for multiple testing. An increase in serum derived C3, IL-6, CD163, and GDF-15 was observed in both nulliparous and parous women, with no significant difference in late values controlled for baseline values (Table 3). No significant change in CRP or ICAM1 was observed in either group from early to late pregnancy after correction for multiple testing. A significant increase in IL-17A and TNF-α was seen only in parous women from early to late pregnancy, while only nulliparous women showed a significant increase in leptin. In addition, nulliparous women had a significantly higher concentration of leptin than parous women in late pregnancy when controlled for early pregnancy values using ANCOVA (Fig. 2A). Absolute differences in biomarker concentrations from early to late pregnancy in each group were also calculated but no significant differences were found (data available upon request). These results suggest that compared to nulliparous women, parous mothers have a higher suppression of cellular immune responses from early to late pregnancy. While both groups show increased levels of serum immune markers with gestation, the magnitude of some of these changes differs with parity states.

**Table 2:** PBMC cytokines and Parity.

		Nulliparous *median (IQR) n = 21*	Parous *median (IQR) n = 51*	p-value
**IL-6**				
LPS	Early	34.64 (1.59, 642.85)	167.74 (10.14, 1385.29)	0.179^c^
	Late	19.03 (6.74, 338.34)	293.73 (14.21, 1068.14)	0.088^d^
	Paired	0.131^a^	0.903^a^	
R848	Early	107.14 (1.46, 2184.98)	397.59 (21.25, 1270.02)	0.410^c^
	Late	34.09 (5.13, 250.62)	269.08 (23.81, 822.01)	0.362^d^
	Paired	0.455^a^	0.708^a^	
**TNF-α**				
LPS	Early	723.03 (25.17, 3740.88)	653.60 (56.34, 6693.83)	0.606^e^
	Late	67.40 (7.85, 2370.31)	225.88 (8.51, 2514.19)	0.029^d^
	Paired	0.958^a^	0.057^a^	
R848	Early	1021.07 (71.09,5663.71)	1042.62 (77.15, 8670.39)	0.714^e^
	Late	179.25 (22.19, 3001.12)	437.54 (20.59, 4115.95)	0.043^d^
	Paired	0.487^b^	**0.018**^b^*	
**IL-10**				
Anti-CD3/28/2	Early	157.38 (16.24, 2256.99)	651.30 (122.74, 2016.03)	0.132^c^
	Late	238.16 (26.22, 626.17)	165.63 (35.38, 937.75)	0.431^d^
	Paired	0.986^a^	**0.011**^a^*	
**IL-2**				
Anti-CD3/28/2	Early	8887.85 (1679.84, 20506.26)	15379.23 (4023.11, 37090.66)	0.218^c^
	Late	6819.62 (1052.10, 47600.79)	5372.52 (1069.58, 35868.98)	0.308^d^
	Paired	0.476^a^	0.606^a^	
**IFN-γ**				
Anti-CD3/28/2	Early	56387.31 (9700.02, 280399.46)	36456.58 (6039.94, 968548.00)	0.926^c^
	Late	7212.62 (761.24, 438120.50)	23109.50 (859.01, 179391.00)	0.570^d^
	Paired	0.357^a^	**0.001**^a^*	

Comparison of fold change values in early and late pregnancy between nulliparous and parous mothers. Parity refers to whether a patient has previously given birth to a neonate. Nulliparous = no previous pregnancies. Parous = had one or more previous pregnancies. n = 72.

^a^Wilcoxon Rank Sum test on abnormally distributed data.

^b^Paired t-test on normally distributed data.

^c^Mann-Whitney U test on abnormally distributed data.

^d^p value generated via Analysis of covariance comparing late pregnancy cytokine values between parity groups controlled for baseline (early) levels.

^e^Independent T-test on normally distributed log-transformed data.

*Significant after Benjamini Hochberg adjustment for multiple comparisons.

**Table 3:** Serum Biomarker Concentrations and Parity.

Marker		n	Nulliparous *median (IQR)*	n	Parous *median (IQR)*	p-value
**C3** *g/l*	Early	21	1.54 (1.46, 1.76)	51	1.60 (1.47, 1.75)	0.631^c^
	Late	21	1.83 (1.69, 1.96)	51	1.82 (1.72, 2.03)	0.591^d^
	Paired		** < 0.001**^a^*		** < 0.001**^a^*	
**CRP** *mg/l*	Early	21	3.58 (1.86, 6.29)	51	2.98 (1.87, 5.25)	0.647^f^
	Late	21	3.17 (2.06, 6.05)	51	3.45 (2.21, 5.25)	0.546^d^
	Paired		0.714^b^		0.159^b^	
**IL-17A** *pg/ml*	Early	21	0.48 (0.28, 0.90)	47	0.55 (0.22, 0.78)	0.947^e^
	Late	19	0.81 (0.46, 1.26)	51	0.82 (0.45, 1.15)	0.991^d^
	Paired		0.030^a^		**0.005**^a^*	
**IL-6** *pg/ml*	Early	20	1.17 (0.78, 1.96)	50	1.11 (0.86, 1.33)	0.161^f^
	Late	21	1.76 (1.50, 2.50)	50	1.43 (1.07, 1.79)	0.197^d^
	Paired		**0.003**^a^*		**0.002**^a^*	
**TNF-α ***pg/ml*	Early	21	7.97 (7.02, 8.63)	50	7.83 (7.36, 8.57)	0.950^e^
	Late	21	8.50 (7.40, 9.75)	51	8.41 (7.47, 9.53)	0.877^d^
	Paired		0.039^a^		**0.003**^a^*	
**CD163** *ng/ml*	Early	21	364.00 (300.26, 486.88)	50	350.84 (310.38, 415.17)	0.659^e^
	Late	21	517.15 (478.68, 628.10)	50	459.50 (363.76, 558.59)	0.191^d^
	Paired		** < 0.001**^a^*		** < 0.001**^a^*	
**ICAM1** *ng/ml*	Early	21	309.37 (289.70, 348.51)	50	322.75 (272.26, 339.90)	0.867^c^
	Late	21	304.81 (289.86, 356.41)	51	281.76 (242.37, 349.88)	0.091^d^
	Paired		0.745^b^		0.034^b^	
**GDF15** *ng/ml*	Early	21	9.77 (7.67, 10.95)	50	9.16 (7.44, 10.79)	0.633^c^
	Late	21	14.38 (12.34, 18.12)	51	14.91 (12.34, 18.19)	0.943^d^
	Paired		** < 0.001**^a^*		** < 0.001**^a^*	
**Leptin** *ng/ml*	Early	21	24.40 (13.97, 39.74)	50	21.50 (16.79, 30.97)	0.536^f^
	Late	21	36.52 (24.61, 47.59)	51	27.39 (15.00, 39.81)	**0.018**^d^*
	Paired		**0.001**^b^*		0.255^b^	

Comparison of serum concentration values in early and late pregnancy between nulliparous and parous mothers. Parity refers to whether a patient has previously given birth to a neonate. Nulliparous = no previous pregnancies. Parous = had one or more previous pregnancies.

^a^Wilcoxon Rank Sum test on abnormally distributed data.

^b^Paired t-test on normally distributed data.

^c^Independent t-test on normally distributed data.

^d^p-value generated via Analysis of covariance comparing late pregnancy marker values between parity groups controlled for baseline (early) levels.

^e^Mann-Whitney U test on abnormally distributed data.

^f^Independent t-test on normally distributed log-transformed data.

*Significant after Benjamini Hochberg adjustment for multiple comparisons.

### Impact of BMI on immune activity

Women with a healthy BMI (18–25 kg/m^2^) showed significant decreases in the fold change of R848 stimulated TNF-α, anti-CD3/28/2 stimulated IL-10, and anti-CD3/28/2 stimulated IFN-γ from early to late pregnancy using paired t-tests or Wilcoxon rank sum tests, while these differences were not seen in women with a BMI ≥ 25 (Table 4). No significant differences were found between groups when comparing late pregnancy fold change values accounting for early pregnancy results using ANCOVA tests. Gestational changes examined through paired t-tests and Wilcoxon rank sum tests in the serum showed a significant increase in C3, IL-6, CD163, and GDF-15 in both BMI groups (Table 5). No change in CRP or ICAM1 throughout pregnancy was observed in either group. Comparison of marker levels at individual timepoints explored through independent t-tests, Mann-Whitney U tests and ANCOVA showed a few notable differences between groups. In contrast to women with a high BMI, women with a healthy BMI showed significantly higher levels of GDF-15 in early pregnancy and had a significant increase in IL-17A throughout pregnancy. Women with higher BMI had significantly higher leptin in early pregnancy, and significantly higher C3 in early and late pregnancy (Fig. 2B). A significant increase in TNF-α throughout gestation was also observed in this group. The relationship between immune markers and BMI was further explored through linear regression using continuous BMI values in both the serum (Supplementary Table S2) and PBMCs (data available upon request). When unadjusted and adjusted for parity, maternal age at recruitment, and Pobal HP Deprivation Index (HP-index), C3 in early pregnancy, and CD163 and Leptin in early and late pregnancy had significant positive associations with BMI. In contrast to this, GDF-15 had a negative association with BMI in early and late pregnancy. Unadjusted C3 in late pregnancy had a positive association with BMI. Significance was lost in late pregnancy after adjustment for multiple comparisons. In the PBMCs, there were no significant associations found with continuous BMI. Absolute differences in biomarker concentrations from early to late pregnancy in each group were also calculated but no significant differences were found (data available upon request). These results indicate that a healthy BMI is associated with a more marked suppression of immune cell activity, and lower levels of inflammatory markers in the serum.

**Table 4:** PBMC cytokines and BMI.

		BMI < 25 *median (IQR) n = 38*	BMI ≥ 25 *median (IQR) n = 34*	p-value
**IL-6**				
LPS	Early	107.51 (7.68, 1514.83)	131.27 (7.80, 893.52)	0.787^c^
	Late	83.57 (5.33, 591.26)	114.79 (12.82, 1226.34)	0.841^d^
	Paired	0.255^a^	0.778^a^	
R848	Early	409.38 (4.45, 1944.87)	230.64 (8.02, 961.20)	0.398^c^
	Late	97.80 (4.30, 673.28)	246.52 (21.82, 638.96)	0.367^d^
	Paired	0.755^a^	0.858^a^	
**TNF-α**				
LPS	Early	826.19 (30.64, 8651.00)	515.70 (50.38, 4964.79)	0.524^e^
	Late	154.40 (7.69, 2542.80)	221.28 (9.33, 2309.60)	0.851^d^
	Paired	0.037^b^	0.206^b^	
R848	Early	1555.79 (100.66, 17398.14)	590.55 (61.93, 5245.85)	0.348^e^
	Late	260.83 (20.45, 2909.52)	345.03 (20.71, 4542.90)	0.572^d^
	Paired	**0.011**^b^*	0.438^b^	
**IL-10**				
Anti-CD3/28/2	Early	679.27 (59.00, 2438.73)	424.80 (24.69, 1758.65)	0.239^c^
	Late	243.95 (34.97, 586.66)	87.08 (27.16, 1070.28)	0.081^d^
	Paired	**0.012**^a^*	0.343^a^	
**IL-2**				
Anti-CD3/28/2	Early	17683.98 (3224.29, 36509.61)	11241.77 (3638.83, 28432.48)	0.580^c^
	Late	2976.10 (640.03, 17695.26)	8177.38 (2042.97, 60204.78)	0.870^d^
	Paired	0.396^a^	0.369^a^	
**IFN-γ**				
Anti-CD3/28/2	Early	47317.85 (9639.67, 336536.14)	42635.79 (5998.96, 1570,107.63)	0.885^e^
	Late	14530.56 (790.08, 104598.60)	16271.66 (805.07, 345040.55)	0.547^d^
	Paired	**0.010**^b^*	0.062^b^	

Comparison of fold change values in early and late pregnancy between mothers with a baseline BMI of less than 25, and mothers with a baseline BMI of greater than or equal to 25. n = 72.

^a^Wilcoxon Signed Rank test on abnormally distributed data.

^b^Paired t-test on normally distributed log transformed data.

^c^Mann-Whitney U test on abnormally distributed data.

^d^*p* value generated via Analysis of covariance comparing PBMC marker fold change levels in late pregnancy between BMI groups controlled for baseline (early) levels.

^e^Independent T-test on normally distributed log-transformed data.

*Significant after Benjamini Hochberg adjustment for multiple comparisons.

**Table 5:** Serum biomarker concentrations and BMI.

Biomarker		n	BMI < 25 *median (IQR)*	n	BMI > = 25 *median (IQR)*	p-value
**C3** *g/l*	Early	38	1.51 (1.38, 1.63)	34	1.71 (1.57, 1.77)	** < 0.001**^c^*
	Late	38	1.75 (1.62, 1.87)	34	1.94 (1.82, 2.11)	**0.020**^d^*
	Paired		** < 0.001**^a^*		** < 0.001**^a^*	
**CRP** *mg/l*	Early	38	2.53 (1.73, 5.12)	34	3.34 (2.21, 5.40)	0.567^e^
	Late	38	3.31 (2.11, 5.08)	34	3.49 (2.15, 6.57)	0.890^d^
	Paired		0.231^b^		0.510^b^	
**IL-17A** *pg/ml*	Early	36	0.51 (0.19, 0.84)	32	0.58 (0.31, 0.78)	0.602^**c**^
	Late	37	0.81 (0.50, 1.21)	33	0.82 (0.44, 1.16)	0.208^d^
	Paired		**0.001**^a^*		0.142^a^	
**IL-6** *pg/ml*	Early	37	0.97 (0.79, 1.24)	33	1.26 (1.01, 1.59)	0.040^c^
	Late	37	1.39 (1.06, 1.71)	34	1.80 (1.31, 2.18)	0.171^d^
	Paired		**0.005**^a^*		**0.001**^a^*	
**TNF-α** *pg/ml*	Early	37	7.86 (7.02, 8.56)	34	7.89 (7.36, 8.61)	0.679^c^
	Late	38	8.08 (7.25, 9.53)	34	8.53 (7.58, 10.23)	0.329^d^
	Paired		0.068^a^		**0.001**^a^*	
**CD163** *ng/ml*	Early	37	322.78 (294.37, 397.75)	34	374.79 (323.25, 458.33)	0.034^e^
	Late	38	452.51 (363.76, 526.81)	33	516.66 (426.68, 637.08)	0.169^d^
	Paired		** < 0.001**^a^*		** < 0.001**^a^*	
**ICAM1** *ng/ml*	Early	37	311.20 (268.49, 353.04)	34	319.70 (295.33, 338.75)	0.448f.
	Late	38	283.64 (243.22, 314.63)	34	316.08 (261.71, 362.34)	0.221^d^
	Paired		0.133^a^		0.912^a^	
**GDF15** *ng/ml*	Early	37	10.60 (8.12, 11.36)	34	8.57 (7.32, 9.78)	**0.016**^c^*
	Late	38	15.64 (11.85, 18.62)	34	14.81 (12.55, 17.84)	0.612^d^
	Paired		** < 0.001**^a^*		** < 0.001**^a^*	
**Leptin** *ng/ml*	Early	37	18.05 (11.82, 24.21)	34	30.31 (21.33, 44.48)	** < 0.001**^c^*
	Late	38	20.15 (11.21, 36.34)	34	35.25 (27.86, 49.61)	0.615^d^
	Paired		**0.005**^a^*		0.185^a^	

Comparison of biomarker concentrations in early and late pregnancy between mothers with a baseline BMI of less than 25, and mothers with a baseline BMI of greater than or equal to 25. n = 72.

^a^Wilcoxon Rank Sum test on abnormally distributed data.

^b^Paired t-test on normally distributed log transformed data.

^c^Mann-Whitney U test on abnormally distributed data.

^d^*p* value generated via Analysis of covariance comparing serum marker concentrations in late pregnancy between BMI groups controlled for baseline (early) levels.

^e^Independent T-test on normally distributed log-transformed data.

^f^Independent T-test on normally distributed data.

*Significant after Benjamini Hochberg adjustment for multiple comparisons.

### Relationship between groups and effect on immune activity

Due to the previously noted associations between BMI and C3, GDF-15, and both parity and BMI with leptin, a multiple linear regression analysis was performed, which accounted for both parity and BMI as predictors of C3, GDF-15 and leptin (Table 6). When accounting for BMI, parity remained associated with late pregnancy leptin with adjusted coefficients similar to the crude mean differences. Similarly, when accounting for parity, BMI remained associated with early pregnancy C3, late pregnancy C3, and early pregnancy leptin. BMI was also related to leptin levels in late pregnancy when controlling for the effect of parity. The association of BMI with GDF-15 was lost when accounting for parity as an independent variable after correction for multiple testing. These results reaffirm the impact of BMI on C3 levels, and both parity and BMI on leptin levels. The effects of BMI on GDF-15 levels appears to hold greater complexity.

**Table 6:** Relationship of selected biomarkers with BMI and Parity.

Dependent variables	Adj. R^2^	Parity			BMI		
		β	95% CI	p-value	β	95% CI	p-value
**C3 early**^a^	0.141	0.005	[− 0.105, 0.115]	0.928	0.184	[0.084, 0.285]	< 0.001*
**C3 late**^b^	0.176	− 0.042	[− 0.167, 0.084]	0.508	0.237	[0.123, 0.351]	< 0.001*
**GDF-15 early**^a^	0.049	− 0.140	[− 1.423, 1.142]	0.828	− 1.358	[-2.526, − 0.191]	0.023
**Leptin early**^c^	0.293	− 0.078	[− 0.190, 0.034]	0.170	0.282	[0.180, 0.384]	< 0.001*
**Leptin late**^c^	0.294	− 0.206	[− 0.326, − 0.085]	< 0.001*	0.265	[0.156, 0.375]	< 0.001*

Linear regression of early and late serum analytes found to be significantly different between groups in Tables 3, 5, controlled for parity and BMI.

*CI* confidence interval, *Early* biomarker measured in early pregnancy, *Late* biomarker measured in late pregnancy.

^a^Normally distributed data.

^b^Abnormally distributed data.

^c^Normally distributed log transformed data.

*Significant after Benjamini Hochberg adjustment for multiple comparisons.

### Impact of a probiotic intervention

As the primary study involved the use of a probiotic intervention, the effects of the intervention group on the serum variables which had significant gestational changes **(**Table 1**)** were investigated (Supplementary Table S3). No differences were noted between probiotic and placebo groups at late pregnancy when accounting for early pregnancy values through ANCOVA, apart from CD163. Analyses of PBMC derived cytokines grouped by intervention were also explored (Supplementary Table S4). Paired t-tests showed that R848 stimulated TNF-α significantly decreased in the intervention group, while anti-CD3/28/2 stimulated IFN-γ significantly decreased in the placebo group. No significant differences in IL-10 were observed. Intervention group had no effect on the significant differences observed between parity and BMI groups (Supplementary Table S5).

## Discussion

### Principal findings

This study demonstrated that the maternal immune system undergoes significant changes from early to late pregnancy which are impacted by parity and BMI. Cellular activity in the peripheral immune system is significantly suppressed from early to late pregnancy. In contrast, immune components of the serum increase. The significance of the gestational changes is impacted by maternal parity and BMI. Having a previous pregnancy and a healthy BMI in early pregnancy are both associated with a more marked dampening of immune cell activity throughout gestation. Parous women also exhibit lower levels of serum leptin at late pregnancy. Women with healthy BMI have lower levels of leptin in early pregnancy, and C3 in early and late pregnancy. Women with healthy BMI also have a more significant increase in IL-17A throughout gestation, and higher levels of GDF-15 in early pregnancy which is a novel finding of this study. The significance of these findings remained when controlling for probiotic usage.

### Results in the context of what is known

Wegmann and colleagues were the first to propose that pregnancy is a state of immunosuppression and tolerance due to a shift from Th1 to Th2 responses^21^. It is now understood that immune modulation in pregnancy displays much greater complexity. Evidence has shown that both pro-inflammatory and anti-inflammatory states are required for successful gestation^22^. Our study of PBMCs in pregnancy supports this, as suppression of PBMC derived TNF-α, IL-10 and IFN-γ was observed from early to late pregnancy. This in line with previous research which reported decreased mRNA and decreased secretion of PBMC derived IL10, TNF-α, and IFN-γ throughout pregnancy in healthy nulliparous and parous patients^9,11^. Reduction in IFN-γ mRNA expression was also reported by Tranchot-Diall et al. in late pregnancy, but in contrast to our findings, levels of TNF-α increased from early pregnancy to month 7, and IL-10 levels fluctuated over time^6^. However, the contrast of this finding to our study may be explained by the measurement of mRNA rather than secreted protein. Another group reported no change in LPS-stimulated IFN-γ, IL-6 or TNF- α, however significant decreases were observed across pregnancy in unstimulated and PHA-stimulated PBMCs for all three cytokines^12^.

Immune mediators in the serum and plasma are well reported. Our data showed a significant increase in both inflammatory (C3, IL-17A, IL-6, TNFα, and leptin) and anti-inflammatory (GDF-15 and CD163) markers throughout pregnancy. Results from other studies are varied. Some groups have reported stable production of inflammatory mediators such as TNF-α and IL-6 throughout pregnancy^7^, while others have shown increased levels of some mediators such as TNF-α and G-CSF, but no change in IL-6^8^. Outside of the commonly reported cytokines, there is a gap in the literature describing further immune mediators. To our knowledge, no study has reported levels of GDF-15 in early and late pregnancy. GDF-15, a cytokine known to limit inflammation^23^, is currently of prime interest in metabolic research as it has been shown to lower body weight and reduce food intake^24^. In our cohort, elevated GDF-15 was observed in mothers with a healthy BMI compared to those with overweight or obesity which suggests that this marker may be important to consider in future studies of metabolic conditions such as gestational diabetes. Across both parity groups and both BMI groups, GDF-15 significantly increased throughout pregnancy.

Few studies have examined both cellular activity and circulating biomarkers in matched samples throughout pregnancy. Our results showed differences between serum and PBMC derived cytokines which could be ascribed to the source of cytokine production. PBMC derived cytokines are produced by stimulated lymphocytes and monocytes and therefore show the capability of a patient’s immune cells to respond to infection^25^. In contrast, serum cytokines may be produced by endothelial cells or fibroblasts in addition to immune cells^26^, and show the circulating levels of cytokine at the time of sampling. Other groups have also noted differences between the two cytokine sources^27^. Expanding upon their previous analysis of serum cytokines in pregnancy, Kraus et al*.* investigated immune cell activities and secreted cytokines in 50 healthy pregnant women. NK and T-cell numbers decreased, as well as secretion of TNF-α, IL-10, IL-6, and IFN-γ after stimulation. Within the same patients, serum inflammatory mediators increased with gestation^9^. However, stimulated cytokine production and circulating cytokines have also been reported to increase during pregnancy^28^.

Conflicting results could be attributed to several factors. There is substantial variability in the timing, number, and type of samples which makes direct comparisons between studies challenging. In addition, maternal and fetal characteristics may also impact results. Mitchell et al. found that levels of TNF-α in early pregnancy, IL-1β in mid and late pregnancy, and IL-6 in early, mid and late pregnancy were significantly higher in women carrying female fetuses^28^, suggesting that fetal sex may impact the maternal immune response. Other factors that could be at play relate to maternal characteristics. Our results showed that the significance of gestational immune changes is impacted by parity and maternal BMI. Parity status has previously been explored as a potential impacting factor, but results are conflicting. Ross et al*.* reported an increase in inflammatory markers TNF-α and IL-6 in healthy nulliparous mothers throughout gestation^4^, while Ferguson et al*.* reported elevated levels of CRP in parous women^5^. Others have reported no relationship between parity and levels of IL-6 and CRP during pregnancy^29^. To our knowledge, no group has examined PBMC responses in relation to parity. High BMI has previously been associated with increased inflammation^30^. This remains true during pregnancy, as shown by several groups in the serum and plasma^4,5,17^.

Finally, ingestion of probiotic foods or supplements may also impact immune responses^31^. Previous research suggests probiotic supplementation may have potential benefits on fertility, maternal health, and infant gut health^32–35^. In healthy pregnant woman, one publication reported that levels of IL-5, IL-6, TNF-α, and GM-CSF significantly increased in comparison to the control group after probiotic administration with *Bifidobacterium longum*, *Lactobacillus delbrueckii bulgaricus* and *Streptococcus thermophilus*^36^. However, in women with co-morbidities such as obesity, this effect may not be seen. In a trial of probiotic supplementation in obese pregnant women, no impact was seen on maternal inflammation^37^. In the present study, CD163 was significantly higher in the probiotic group in late pregnancy, suggesting a potential anti-inflammatory effect. Additionally, from early to late pregnancy, a more significant increase in serum TNFα and IL17A, and a significant decrease in PBMC derived IFN-γ, was observed in mothers who received probiotic supplementation, suggesting that probiotics may attenuate the gestational changes to maternal measured serum immune responses.

### Clinical implications

The contrasting activity of PBMC cytokines and serum markers suggests that serum analyses do not provide a complete picture of a person’s immune profile^27^. PBMC analysis may be beneficial for predicting a patient’s ability to respond to infection or combat disease. Therefore, more comprehensive analysis on whole blood samples may be required in some patients. The differences identified within serum samples between parity and BMI groups at individual time points in pregnancy likely have no clinical significance due to the marginal differences in marker concentrations in each group. However, these factors do affect the gestational changes that occur in patients and may be informative of the immune trajectories of mothers with nulliparity and high BMI. Additionally, we have shown differences in immune responses between nulliparous and parous women (Tables 2, 3), which may explain why parous women are less likely to experience placental disease, such as pre-eclampsia and fetal growth restriction.

### Strengths and limitations

This study has several strengths. Every PBMC derived cytokine in our analysis was detectable. Our study provides insight into the activity of a robust panel of immune markers in both PBMCs and serum, including GDF-15, ICAM1 and CD163 which have been scarcely reported longitudinally throughout pregnancy before. Our study also provides evidence linking parity to both cellular and serum immune activity, of which scant literature exists.

Our study is not without limitations. Not all serum immune markers were detectable and therefore were excluded from analyses. The higher number of parous patients may have underrepresented the immune activity of nulliparous women. Due to the primary study design, our analysis was limited to two timepoints in pregnancy. As this is a secondary analysis, the outcomes of this study were not powered, and the small sample size may limit the applicability of the results on a global scale. As pre-pregnancy BMI data was unavailable, BMI was measured in early pregnancy. BMI as a measure of adiposity has been critiqued by some groups due to its lack of accountability for muscle mass. There was no adjustment for other potential confounders besides parity and BMI.

## Conclusions

The peripheral immune system undergoes significant changes throughout pregnancy. Immune activity is dampened in peripheral immune cells while immune components of the serum are heightened. These differences are altered by parity and BMI and aid our understanding as to why first-time mothers are at greater risk of placental disease such as pre-eclampsia and fetal growth restriction.

## Methods

### Study design and population

This a secondary analysis of 72 healthy pregnant women who participated in a double blind randomized controlled trial (MicrobeMom2 RCT) of maternal probiotic supplementation with *Bifidobacterium longum* subsp. *longum* 1714® versus placebo. This study was conducted in the National Maternity Hospital, Dublin, Ireland from 2020 to 2022 in accordance with the guidelines of the Declaration of Helsinki, with ethical approval granted by the National Maternity Hospital Research Ethics Committee. Informed consent was obtained from all subjects and/or their legal guardian(s). The methods have been published elsewhere^19^. Briefly, women were recruited during their first antenatal visit at 11–15 weeks gestation. Inclusion criteria were singleton pregnancy, maternal age 18 to 45 years, BMI between 18.5 and 35 kg/m^2^, capable of giving informed consent, and sufficient English language to allow for study comprehension. Exclusion criteria included multiple pregnancy, previous miscarriage, fetal anomaly, previous or current gestational diabetes (GDM), diabetes mellitus or pre-diabetes, other medical conditions requiring medical treatment, or an unwillingness to limit the consumption of supplements or probiotic food during the trial. Participants were randomly allocated to intervention or control groups using block randomisation. Each participant provided two blood samples: one at their initial visit (early pregnancy, 11–15 weeks’ gestation) and another following ≥ 12 weeks of probiotic or placebo supplementation (late pregnancy, 28–32 weeks’ gestation).

### Data collection

Demographic information including maternal age, ethnicity, and parity were collected at the first visit through electronic medical records or self-report. A woman was defined as parous if she had one of more previous term pregnancies. Maternal weight was measured during early pregnancy (11–15 weeks’ gestation) with the mother in light clothing using a SECA weighing scales (SECA GmbH & co. KG. Hamburg, Germany) and recorded to the nearest 0.1 kg. Height was measured after removal of footwear using a wall mounted stadiometer and was recorded to the nearest 0.1cm. Subsequently, maternal BMI was calculated in accordance with the WHO guidelines which define a healthy weight as having a BMI of 18–24.9 kg/m^2^, and overweight or obesity as having a BMI ≥ 25 kg/m^2^^20^. Maternal economic advantage was assessed using the Pobal Haase-Pratschke (HP Pobal) Deprivation Index, which measures relative advantage or disadvantage of the location of residence using Irish census data^38^.

### Sample collection and processing

Peripheral blood samples were collected in VACUETTE® K2EDTA tubes for the purpose of PBMC isolation. These methods have been described previously^39^. Briefly, PBMCs were isolated from whole blood by density gradient centrifugation and frozen until use. Subsequently, cells were thawed and stimulated with LPS, R848, and anti-CD3/28/2. Detailed methodology is available in Supplementary Information S6. Serum samples were collected in VACUETTE® serum tubes (Greiner Bio-One, Austria) at early and late pregnancy. Serum was isolated from the peripheral whole blood by centrifugation on a Rotina 420R (Hettich, MA, USA) at 3000 rpm for 10 min with the break and accelerator set to 3 and stored at −80 ℃ until use. Serum concentrations of CRP and C3 were determined through the Cobas® system (Roche, Basel), while levels of IL-17A, IL-6, TNF-α, CD163, ICAM1, GDF-15, and leptin were analysed by the ProteinSimple ELLA automated ELISA immunoassay system (Biotechne, Minneapolis) according to manufacturer’s instructions.

### Statistical analysis

Statistical analyses were performed using IBM SPSS version 27.0 for Windows (SPSS Inc, Chicago, IL). Normality of the data was determined through the Shapiro-Wilk test, examination of histograms, and inspection of descriptive information. Skewed data underwent log^10^ transformation and was again assessed for normality before carrying out the appropriate parametric or non-parametric test. Continuous variables are presented as mean (standard deviation) for normally distributed data, or median (25th, 75th percentile) for skewed data. Stimulated cytokine values are reported as fold change from unstimulated to stimulated state, while serum cytokine values are reported in g/l (C3), mg/l (CRP), pg/ml (IL-17A, IL-6, TNFα), or ng/ml (CD163, ICAM1, GDF-15 and Leptin). Gestational changes in biomarkers were evaluated through paired t-tests or Wilcoxon signed rank tests. Differences in concentrations between groups in early pregnancy were assessed through independent t-tests or Mann-Whitney U tests. Differences in late pregnancy values were assessed through an analysis of covariance (ANCOVA) between groups, controlled for the early value as a covariate. Significantly different variables underwent linear regression analysis to assess if the grouping factor remained predictive while accounting for both factors simultaneously. Linear regression analysis with BMI as a continuous independent variable was also conducted to further explore the relationship between BMI and serum immune markers. The effect of the primary study intervention group on parity or BMI associated differences was investigated through an ANCOVA by using the intervention group as a covariate. To inspect potential effects of the probiotic on gestational immune changes, participants were split by intervention group, and paired t-tests or Wilcoxon signed rank tests were repeated. The Benjamini-Hochberg method was used to adjust the data for multiple comparisons using an FDR of 0.1.

### Supplementary Information

Supplementary Information.

## Data Availability

The datasets used and analyzed during the current study are available from the corresponding author upon reasonable request.
