# Supplementary material for: Impact of previous pregnancy and BMI on cellular and serum immune activity from early to late pregnancy
Source: Sci Rep. 2024 Jul 11;14:16055. doi: 10.1038/s41598-024-66651-4 (PMC11239859; doi:10.1038/s41598-024-66651-4)
Supplement: Supplementary file 1 — Supplementary Information. [file 41598_2024_66651_MOESM1_ESM.docx]

# Supplementary Materials

**Supplementary Table S1:** PBMC Cytokine Concentrations from Early to Late Pregnancy in pg/ml

**Supplementary Table S2:** Relationship between serum biomarkers and BMI (continuous)

**Supplementary Table S3:** Change in Serum Marker Concentrations from Early to Late Pregnancy

**Supplementary Table S4:** PBMC cytokines from early to late pregnancy by intervention group

**Supplementary Table S5:** Differences in biomarker concentrations between BMI groups controlled for intervention group

**Supplementary Information S6:** PBMC Processing and Analysis

| **Supplementary Table S1. PBMC Cytokine Concentrations from Early to Late Pregnancy in pg/ml** | | | | |
| --- | --- | --- | --- | --- |
| **Unstimulated** |  |  |  |  |
|  |  | **Early Pregnancy** *Median pg/ml (IQR)* | **Late Pregnancy** *Median pg/ml (IQR)* | **P-value** |
| **IL-6** | **-** | 18.81 (5.07, 668.33) | 10.29 (1.62, 81.77) | **<0.001*** |
| **TNFα** | - | 2.28 (0.72, 5.85) | 1.03 (0.41, 2.43) | **<0.001*** |
| **IL-10** | - | 0.78 (0.41, 2.91) | 0.50 (0.29, 1.05) | **<0.001*** |
| **IL-2** | - | 0.30 (0.11, 0.59) | 0.20 (0.10, 0.38) | **<0.001*** |
| **IFNγ** | - | 0.58 (0.00, 2.52) | 0.92 (0.00, 1.78) | 0.964 |
|  |  |  |  |  |
| **Stimulated** |  |  |  |  |
|  | **Stimulation** | **Early Pregnancy** *Median pg/ml (IQR)* | **Late Pregnancy** *Median pg/ml (IQR)* | **P-value** |
| **IL-6** | LPS (24hr) | 7818.02 (2400.18, 17643.79) | 2195.75 (528.21, 6463.54) | **<0.001*** |
|  | R848 (24hr) | 8253.47 (2703.35, 18766.06) | 2548.73 (458.76, 9342.45) | **<0.001*** |
| **TNFα** | LPS (24hr) | 4376.05 (904, 7798.78) | 92.13 (7.18, 5025.08) | **<0.001*** |
|  | R848 (24hr) | 5821.86 (1329.35, 12558.04) | 114.89 (19.09, 7144.34) | **<0.001*** |
| **IL-10** | Anti-CD3/28/2 (48hr) | 738.81 (251.44, 1457.88) | 119.45 (10.56, 766.05) | **<0.001*** |
| **IL-2** | Anti-CD3/28/2 (48hr) | 3181.66 (1139.42, 6563.01) | 1230.01 (216.29, 4375.09) | **0.009*** |
| **IFNγ** | Anti-CD3/28/2 (48hr) | 23927.74 (9291.14, 53448.11) | 4904.53 (868.9, 21856.02) | **<0.001*** |
| Supplementary Table S1 shows the concentration (pg/ml) of secreted cytokines from PBMCs in both the unstimulated state, and after stimulation with either LPS, R848 or Anti-CD3/28/2. Concentrations of IL-6 and TNFα from stimulated cells were obtained after treatment with LPS and R848 for 24 hours. Concentrations of IL-10, IL-2 and IFN**γ** from stimulated cells were obtained after treatment with Anti-CD3/28/2 for 48 hours. As all data was abnormally distributed, the Wilcoxon Signed Rank Test was used to examine the differences in cytokine secretion from early to late pregnancy. * Significant after Benjamini Hochberg adjustment for multiple comparisons. | | | | |

| **Supplementary Table S2. Relationship between serum biomarkers and BMI (continuous)** | | | | | | | | |
| --- | --- | --- | --- | --- | --- | --- | --- | --- |
|  | | | | | | | | |
|  |  | **Model 1** | | |  | **Model 2** | | |
| **Dependent Variables** | **Adj. R^2^** | **β** | **95% CI** | **p-value** | **Adj.**  **R^2^** | **β** | **95% CI** | **p-value** |
| C3 Early^a^ | 0.181 | 0.026 | 0.013, 0.039 | **<0.001*** | 0.147 | 0.027 | 0.013, 0.040 | **<0.001*** |
| C3 Late^b^ | 0.065 | 0.019 | 0.003, 0.035 | **0.018*** | 0.029 | 0.019 | 0.002, 0.036 | 0.031 |
| CRP Early^c^ | 0.044 | 0.021 | 0.001, 0.042 | 0.042 | 0.010 | 0.022 | 0.000, 0.044 | 0.050 |
| CRP Late^c^ | 0.025 | 0.016 | -0.003, 0.035 | 0.097 | 0.085 | 0.011 | -0.008, 0.030 | 0.259 |
| IL17A Early^b^ | -0.015 | -0.003 | -0.039, 0.033 | 0.864 | -0.020 | -0.012 | -0.049, 0.025 | 0.511 |
| IL17A Late^b^ | -0.005 | -0.014 | -0.047, 0.020 | 0.423 | -0.050 | -0.015 | -0.052, 0.022 | 0.423 |
| IL6 Early^c^ | 0.045 | 0.013 | 0.000, 0.025 | 0.043 | 0.164 | 0.018 | 0.006, 0.030 | 0.005 |
| IL6 Late^c^ | 0.048 | 0.011 | 0.001, 0.022 | 0.036 | 0.133 | 0.010 | 0.000, 0.021 | 0.059 |
| TNFα Early^b^ | -0.013 | -0.014 | -0.101, 0.074 | 0.753 | 0.012 | 0.014 | -0.077, 0.104 | 0.764 |
| TNFα Late^b^ | 0.020 | 0.109 | -0.031, 0.248 | 0.124 | -0.003 | 0.134 | -0.014, 0.281 | 0.075 |
| CD163 Early^b^ | 0.099 | 11767.181 | 3796.474, 19737.888 | **0.004*** | 0.068 | 12505.519 | 4027.899, 20983.139 | **0.004*** |
| CD163 Late^c^ | 0.018 | 0.006 | -0.002, 0.015 | 0.134 | 0.026 | 0.006 | -0.003, 0.015 | 0.168 |
| ICAM1 Early^a^ | -0.005 | 1376.368 | -2090.179, 4842.914 | 0.431 | 0.050 | 2405.485 | -1118.118, 5929.088 | 0.178 |
| ICAM1 Late^a^ | -0.009 | 1276.756 | -3013.841, 5567.352 | 0.555 | 0.007 | 1416.553 | -3040.809, 5873.914 | 0.528 |
| GDF-15 Early^a^ | 0.137 | -253.451 | -398.772 , -108.130 | **<0.001*** | 0.147 | -218.631 | -369.672, -67.590 | **0.005*** |
| GDF-15 Late^b^ | -0.002 | -173.714 | -544.004, 196.577 | 0.353 | -0.034 | -223.590 | -617.619, 170.438 | 0.261 |
| Leptin Early^c^ | 0.490 | 0.047 | 0.035, 0.058 | **<0.001*** | 0.482 | 0.047 | 0.035, 0.059 | **<0.001*** |
| Leptin Late^c^ | 0.245 | 0.036 | 0.022, 0.051 | **<0.001*** | 0.317 | 0.039 | 0.024, 0.053 | **<0.001*** |
|  | | | | | | | | |
| Linear regression of BMI (continuous) and early and late serum analytes. Model 1 is unadjusted. Model 2 is adjusted for parity, maternal age at recruitment, and HP-index. ^a^Normally distributed data. ^b^Abnormally distributed data. ^c^Normally distributed log transformed data. *Significant after Benjamini Hochberg adjustment for multiple comparisons. CI = Confidence interval. Early = Biomarker measured in early pregnancy. Late = Biomarker measured in late pregnancy. | | | | | | | | |

| **Supplementary Table S3. Change in Serum Marker Concentrations from Early to Late Pregnancy** | | | | | | |
| --- | --- | --- | --- | --- | --- | --- |
|  | | | | | | |
| **Marker** |  | ***n*** | **Probiotic** *Median (IQR)* | ***n*** | **Placebo** *Median (IQR)* | **p-value** |
|  |  |  |  |  |  |  |
| C3  (g/l) | Early | 36 | 1.56 (1.47, 1.74) | 36 | 1.60 (1.45, 1.76) | 0.427^c^ |
|  | Late | 36 | 1.81 (1.67, 2.00) | 36 | 1.88 (1.75, 2.12) | 0.281^d^ |
|  | Paired T-test^a^ |  | **<0.001*** |  | **<0.001*** | - |
| IL-17A (pg/ml) | Early | 36 | 0.37 (0.17, 0.74) | 32 | 0.64 (0.49, 0.99) | **0.006^c*^** |
|  | Late | 34 | 0.74 (0.39, 1.25) | 36 | 0.87 (0.52, 1.14) | 0.942^d^ |
|  | Paired T-test^a^ |  | **0.004*** |  | 0.033 | - |
| IL-6 (pg/ml) | Early | 35 | 1.14 (0.81, 1.49) | 35 | 1.12 (0.82, 1.47) | 0.751^e^ |
|  | Late | 35 | 1.56 (1.37, 2.00) | 36 | 1.53 (1.09, 1.90) | 0.514^d^ |
|  | Paired T-test^b^ |  | **<0.001*** |  | **0.004*** | - |
| TNFa (pg/ml) | Early | 36 | 7.77 (6.95, 8.77) | 35 | 7.97 (7.48, 8.50) | 0.836^c^ |
|  | Late | 36 | 8.48 (7.43, 9.68) | 36 | 8.32 (7.45, 9.53) | 0.129^d^ |
|  | Paired T-test^a^ |  | **0.002*** |  | 0.052 | - |
| CD163 (ng/ml) | Early | 36 | 316.03 (283.34, 391.98) | 35 | 378.83 (323.47, 456.94) | **0.008^c*^** |
|  | Late | 36 | 503.22 (384.46, 589.59) | 35 | 473.39 (382.94, 589.26) | **0.010^d*^** |
|  | Paired T-test^a^ |  | **<0.001*** |  | **<0.001*** | - |
| GDF15 (ng/ml) | Early | 36 | 9.28 (8.16, 10.82) | 35 | 8.94 (7.20, 11.16) | 0.520^c^ |
|  | Late | 36 | 14.78 (12.74, 16.91) | 36 | 15.64 (10.94, 18.43) | 0.869^d^ |
|  | Paired T-test^a^ |  | **<0.001*** |  | **<0.001*** | - |
| Leptin (ng/ml) | Early | 36 | 20.85 (13.54, 34.03) | 35 | 23.95 (15.52, 32.43) | 0.648^e^ |
|  | Late | 36 | 28.34 (15.06, 41.72) | 36 | 30.71 (17.02, 43.60) | 0.264^d^ |
|  | Paired T-test^b^ |  | 0.164 |  | **0.014*** | - |
|  |  |  |  |  |  |  |
| Gestational changes in biomarker concentrations assessed through comparing early pregnancy concentrations, late pregnancy concentrations, and changes in concentrations from early to late pregnancy between intervention groups. Only biomarkers which showed significant differences between groups in terms of paired change and biomarker concentration at early and late pregnancy (table 4) are shown.  ^a^*p* value generated via non-parametric paired t-test of abnormally distributed data or  ^b^paired t-test of normally distributed log transformed data assessing the change from early to late pregnancy. *^c^p* value generated via Mann Whitney U or ^e^Independent T-test comparing serum marker concentrations in early pregnancy between intervention groups. ^d^ *p* value generated via Analysis of covariance comparing serum marker concentrations in late pregnancy between groups controlled for baseline (early) levels. *Significant after Benjamini Hochberg adjustment for multiple comparisons. | | | | | | |

| **Supplementary Table S4. PBMC cytokines from early to late pregnancy by intervention group** | | | | | |
| --- | --- | --- | --- | --- | --- |
|  | | | | | |
| **TNF-α** |  | **Probiotic** *median (IQR) n=36* | | **Placebo** *median (IQR) n=36* | **p-value** |
| *LPS* | Early | 946.13 (156.18, 6359.27) | | 644.47 (10.60, 4504.15) | 0.094^c^ |
|  | Late | 344.22 (11.60, 3395.97) | | 117.48 (3.68, 2066.15) | 0.781^d^ |
|  | Paired T-test | 0.023^a*^ | | 0.230^a^ | - |
|  | | | | | |
| *R848* | Early | 1299.07 (174.36, 15187.17) | | 828.58 (15.76, 5363.25) | 0.106^c^ |
|  | Late | 998.60 (20.80, 4599.93) | | 152.06 (21.49, 2297.00) | 0.678^d^ |
|  | Paired T-test | **0.013^a*^** | | 0.348^a^ | - |
| **IL10** | | |  | | |
| *Anti-CD3/28/2* | Early | 540.25 (55.04, 2777.77) | | 519.89 (40.13, 1457.26) | 0.536^e^ |
|  | Late | 189.69 (25.96, 866.57) | | 148.74 (31.67, 887.03) | 0.506^d^ |
|  | Paired T-test | 0.116^b^ | | 0.046^b^ | - |
| **IFN-γ** | | |  | | |
| *Anti-CD3/28/2* | Early | 58756.04 (6859.63, 1131961.75) | | 34502.18 (9906.16, 240752.35) | 0.936^c^ |
|  | Late | 17057.81 (538.66, 357707.25) | | 13744.41 (1213.40, 124230.14) | 0.150^d^ |
|  | Paired T-test | 0.042^a^ | | **0.013^a*^** | - |
| ^a^Paired t-test or ^b^non-parametric equivalent (Wilcoxon signed rank test) comparing early and late fold change values within each group. ^c^ *p* value generated via Independent t-test or ^e^Mann Whitney U test of early pregnancy cytokine values between the intervention groups. ^d^ *p* value generated via Analysis of covariance comparing late pregnancy cytokine values between intervention groups controlled for baseline (early) levels. Early refers to baseline or pre-intervention (11-15 weeks’ gestation). Late prefers to late pregnancy (28-32 weeks’ gestation). *Significant after Benjamini Hochberg adjustment for multiple comparisons. *Significant after Benjamini Hochberg adjustment for multiple comparisons. n=72. Lipopolysaccharide=LPS, Interleukin-6=IL-6 and Tissue Necrosis Factor alpha=TNF-α. | | | | | |

|  | **Supplementary Table S5. Differences in biomarker concentrations between BMI groups controlled for intervention group** | | | | | | | | | | | | |
| --- | --- | --- | --- | --- | --- | --- | --- | --- | --- | --- | --- | --- | --- |
|  |  | | | | | | | | | | | | |
| **Marker** | | | **Probiotic** *Median (IQR)* | | | |  | **Placebo** *Median (IQR)* | | | | **BMI Effect** | **Group Effect** |
|  |  |  | *n* | **BMI <25** | *n* | **BMI >=25** |  | *n* | **BMI <25** | *n* | **BMI >=25** |  |  |
| C3 (g/l) | | Early | 22 | 1.50 (1.37, 1.58) | 14 | 1.72 (1.6, 1.77) |  | 16 | 1.55 (1.35, 1.66) | 20 | 1.70 (1.51, 1.81) | **0.001*** | 0.928 |
|  |  | Late | 22 | 1.71 (1.62, 1.84) | 14 | 1.95 (1.82, 2.04) |  | 16 | 1.77 (1.58, 1.99) | 20 | 1.94 (1.82, 2.13) | **<0.001*** | 0.471 |
| GDF15 (ng/ml) | | Early | 22 | 10.43 (8.67, 11.08) | 14 | 8.57 (7.89, 9.4) |  | 15 | 9.77 (7.47, 12.44) | 20 | 8.53 (7.06, 10.25) | **0.024*** | 0.942 |
| Leptin (ng/ml) | | Early | 22 | 17.7 (11.22, 21.88) | 14 | 37.06 (26.62, 53.51) |  | 15 | 20.67 (12.76, 25.83) | 20 | 25.73 (19.46, 41.71) | **<0.001*** | 0.715 |
|  | |  |  |  | | |  |  |  |  |  |  |  |
|  | |  | *n* | **Nulliparous** | *n* | **Parous** |  | *n* | **Nulliparous** | *n* | **Parous** | **Parity** **Effect** | **Group Effect** |
| Leptin (pg/ml) | | Late | 14 | 36.89 (22.28, 45.38) | 22 | 21.06 (11.05, 33.9) |  | 7 | 36.51 (30.28, 60.32) | 29 | 28.01 (16.72, 42.14) | **0.007*** | 0.135 |
|  | |  |  |  |  |  |  |  |  |  |  |  |  |
| ANCOVA comparing biomarker concentrations of BMI groups controlled for intervention group. Early refers to baseline or pre-intervention (11-15 weeks’ gestation). Late refers to late pregnancy (28-32 weeks’ gestation). *Significant after Benjamini Hochberg adjustment for multiple comparisons. n=72. | | | | | | | | | | | | | |

**Supplementary Information S6. PBMC Processing and Analysis**

PBMCs were isolated from whole blood samples by density gradient centrifugation using Lymphoprep^TM^ (Stemcell, Vancouver) following manufacturer’s instructions. Isolated PBMCs were frozen in FBS containing 10% DMSO and stored in liquid nitrogen until use. Subsequently, cells were thawed and seeded at a density of 2x10^6^ cells/ml in u-bottoms 96-well plates and cultured at 37^°^C, 5% CO2, 95% air in RPMI-1640 with 2.5 Mm L-glutamine and 0.5 Mm sodium pyruvate with 10% FBS (all from Sigma Aldrich, Missouri). To examine both innate and adaptive responses, PBMCs were stimulated with Lipopolysaccharide (LPS)(100ng/mg) (Enzo, Farmingale NY), a TLR4 activator, Resiquimod (R848) (1µg/ml) (Invivogen, San Diego, CA) a TLR7/8 activator, or ImmunoCult^TM^ CD3/CD28/CD2 T-cell activator (Anti-CD3/28/2)(Stemcell, Vancouver). Levels of IL-10, TNF-α, IFN-γ, IL-6 and IL-2 were determined through the BioLegend LEGENDplex^TM^ Human Inflammation Panel (5-plex) performed according to manufacturer’s instructions to reveal the inflammatory (TNF-α, IFN-γ, IL-6, IL-2), and anti-inflammatory (IL-10) potential of cells. Samples were acquired on a BD FSR Fortessa cell analyser and analysis was completed using the BioLegend LEGENDplex^TM^ software. Cytokine values are reported as fold change from unstimulated to stimulated state.
